# Smoked sausages of bovine meat produced in North Macedonia as a source of pro-technological lactic acid bacteria and coagulase-negative cocci

**DOI:** 10.1016/j.heliyon.2024.e37548

**Published:** 2024-09-06

**Authors:** Giorgia Rampanti, Daniela Nikolovska Nedelkoska, Tatjana Kalevska, Tanja Stojanovska, Joanna Harasym, Federica Cardinali, Agnieszka Orkusz, Vesna Milanović, Cristiana Garofalo, Alessio Bonifazi, Lucia Aquilanti, Andrea Osimani

**Affiliations:** a Dipartimento di Scienze Agrarie, Alimentari ed Ambientali, Università Politecnica delle Marche, via Brecce Bianche, 60131, Ancona, Italy; b University “St. Kliment Ohridski” - Bitola, Faculty of Technology and Technical Sciences, Dimitar Vlahov 57, 1400, Veles, North Macedonia; c Department of Biotechnology and Food Analysis, Wroclaw University of Economics and Business, Komandorska 118/120, 53-345, Wrocław, Poland

**Keywords:** Smoked sausages, *Latilactobacillus sakei*, *Staphylococcus equorum*, Protease, Acidification performance, Nitrate reductase

## Abstract

Smoked bovine sausages, traditional meat products from the Balkan Peninsula, are rich in microbial diversity and represent potential sources of pro-technological microorganisms. This study aimed to characterize these sausages from three different producers collected in green markets of North Macedonia. The analyses included physico-chemical (proximate composition, pH, a_w_), morpho-textural (color and texture), and microbiological assessments (viable plate counts). Moreover, an isolation campaign was conducted to identify and characterize pro-technological microorganisms. Significant variability was observed in moisture content (ranging from 33.70 to 48.61 %), hardness, and color among samples from different producers. Samples from producer 2 showed the lowest pH (mean ∼4.90) and the highest loads of lactic acid bacteria (up to ∼9 log cfu g^−1^). Coagulase-negative cocci ranged between 4.84 and 7.47 log cfu g^−1^. No potential pathogenic bacteria were detected. A total of 30 isolates, primarily *Latilactobacillus sakei*, *Staphylococcus equorum*, and *Staphylococcus casei*, were identified. Isolates of L. *sakei* S7, S13, and S27 showed strong in-vitro acidification performance, together with the production of exopolysaccharides (EPS), and protease activity. *S. equorum* isolates S1 and S2 exhibited protease and lipase activities, while isolates *S. casei* S21 and S28 showed notable lipase and protease activities, along with the production of EPS. Additionally, all *S. equorum* isolates, except S2, showed nitrate reductase activity, one of the key features able to affect sausage color. These findings highlighted the pro-technological traits of these microbial isolates, suggesting their potential use as starter or adjunct cultures in the meat industry to enhance product quality and safety.

## 1 Introduction

To date, there is a growing interest in traditional foods that represent a meaningful component of dietary habits and cultural identity of human communities. Of note, in rural areas, culinary heritage is the result of collective memories and origins that foster a sense of belonging to a specific territory [1]. The European continent is characterized by different geographical regions (e.g., the Mediterranean Region, the Balkan Peninsula, the Baltic Region, etc.) that allow the production of several food delicacies, whose characteristics are strongly influenced by specific climatic and environmental conditions.

Traditional foods produced in the Balkan Peninsula represent a magnificent synthesis of various ethnic groups, languages, and traditions [2]. The food culture in the Balkan region is built on several pillars: cereal-based products, milk and dairy products, and meat products [2]. This area is known for a variety of food specialties, including phyllo bread börek (and bajanik) [3], the cereal-based beverage boza [4], the traditional salty Bieno cheese [5], as well as dry-cured sheep meat preparations such as Pastrma, Stelja, and Kastradina [6], and dry beef sausages like Sjenički sudžuk [7].

Regarding meat products, the raw material (fresh meat) is a fragile food matrix that is prone to a rapid decay, due to enzymatic autolysis and microbial activity [8]. Indeed, during the slaughtering of animals, meat can easily be contaminated by environmental and/or intestinal microorganisms, these latter occurring as a result of accidental rupture of the viscera [9]. Among the most detected spoilage or pathogenic microorganisms in raw meat, those of the genera *Acinetobacter*, *Brochothrix*, *Pseudomonas*, *Psychrobacter*, *Salmonella*, *Klebsiella*, *Shigella*, *Yersinia*, and *Escherichia* are included [9]. In order to extend the shelf-life of raw meat, fermentation, smoking, and drying (applied individually or in combination) represent from long time the most suitable methods. Fermented sausages are usually realized with a minced meat (swine, bovine, poultry, or sheep) batter added with lard, salt, and spices, and then stuffed into animal casings [10]. During fermentation, the meat batter of the sausages is subjected to physical and biochemical modifications carried out by meat native enzymes together with autochthonous pro-technological microbial populations. Indeed, once the meat batter is processed, spoilage and pathogenic bacteria are progressively replaced by those with pro-technological function, mainly constituted by lactic acid bacteria and coagulase-negative cocci. Lactic acid bacteria, including *Lactiplantibacillus plantarum*, *Latilactobacillus curvatus*, *Lacticaseibacillus casei*, *Lactiplantibacillus pentosus*, *Pediococcus pentosaceus*, and *Pediococcus acidilactici*, are mainly responsible for lowering the pH by fermenting sugars and producing lactic acid. Meanwhile, coagulase-negative cocci, including *Staphylococcus xylosus*, *Staphylococcus carnosus*, and *Staphylococcus equorum*, contribute to the development of flavor and color through their lipase, protease, and nitrate reductase activities [11].

For smoked sausages, the stuffed meat batter is subjected to smoking under specific environmental conditions, leading to physicochemical and microbiological modifications that define the firmness, cohesiveness, taste, and safety of the final product [12]. Whether smoking is used for preservation or to enhance sensory attributes, it can be performed at either low (cold smoking) or high (hot smoking) temperatures. During cold smoking, the smoke temperature should ideally remain below 20 °C, typically ranging from 15 to 25 °C, and should not exceed 30 °C [13]. In contrast, traditional hot smoking involves reaching smoke temperatures of up to 130 °C and meat temperatures of 80 °C, although some sources suggest lower temperatures, typically ranging between 55 and 80 °C [13]. In smoked sausages, the process of smoking can impact the composition of microbiota, with variables such as temperature and time playing a crucial role. For instance, Kameník et al. [14] demonstrated that *Weissella viridescens* cultures could endure heat treatments at 50 °C and persist within sausages cooked in smoking chambers at 78 °C for 10 min, achieving a core temperature of 70 °C. Similarly, Modzelewska-Kapituła & Maj-Sobotka [15] have noted the presence of *Listeria monocytogenes* in smoked sausage samples from Poland, albeit in small quantities. Despite the potential for smoking treatments to substantially reduce microbial loads, whether through hot or cold smoking, surviving bacteria may rebound post-smoking and proliferate during sausage shelf-life. Consequently, it is conceivable that the naturally occurring microbiota, resilient to smoking processes, could significantly influence the quality of the end product. Awareness and control of these common bacteria during processing are essential for preserving microbiological quality, sensory attributes, and food safety [16]. Additionally, as reported by Dušková et al. [17] and Belleggia et al. [12], artisan smoked sausages (non-fermented salami) are likely to be a source of resilient pro-technological microorganisms. These indigenous microorganisms, well-adapted to the meat environment, can effectively compete with contaminant ones [18]. Such microorganisms can be selected for their use in the meat industry as adjunct cultures (e.g., protective cultures) or starter cultures for fermented meat products. This strategy may help to reduce safety risks and standardize products while maintaining their unique sensory qualities [19].

Based on the above premises, this study investigated the microbiological characteristics of smoked bovine sausages collected in green markets of North Macedonia. Selective growth media were used to assess, for the first time, the potential of these sausages as a reservoir of pro-technological microorganisms. Additionally, physicochemical characteristics were analyzed. The obtained microbial isolates (lactic acid bacteria and coagulase-negative cocci) were identified and characterized for their potential pro-technological features. The characterization included: i) acidification performance (for lactic acid bacteria); ii) nitrate reductase activity (for coagulase-negative cocci); iii) key enzymatic activities (esterase, lipase, and protease); and iv) exopolysaccharides synthesis (EPS).

Of note, sausages can be a source of *Listeria monocytogenes* [9,20], hence, the lactic acid bacteria and coagulase-negative cocci isolates were also tested for the production of bacteriocins against *Listeria innocua*, used as a surrogate for *L. monocytogenes* [21].

Starter or adjunct cultures should be tested for safety characteristics to protect consumers. In more detail, Gram-positive bacteria can be a threat for consumers due to the production of biogenic amines in foods [10]. In Gram-positive bacteria, the production of histamine, that is the causative agent of the scombroid poisoning, is encoded by a gene cluster that includes the *hdcA* gene [10]. Hence, the isolates herein studied were also screened for presence of the *hdcA* gene encoding for histidine decarboxylase.

## 2 Materials and methods

### 2.1 Sampling

Marketed smoked sausages of bovine meat (∼300 g each) were collected from three artisanal butchers (designated as P1, P2, and P3) located in green markets of Skopje (Republic of North Macedonia). For each producer, three batches (namely B1, B2, and B3), for a total of nine samples (namely P1B1, P1B2, P1B3, P2B1, P2B2, P2B3, P3B1, P3B2, and P3B3), were collected and analyzed. All the smoked sausages were prepared using beef, beef fat, salt, nitrate/nitrite, sugar, garlic, red pepper, and other spices in various proportions.

In more detail, for sausages produced by P1, chilled or frozen beef meat and fat were ground on a chopper and then transferred to a tumbler where spices and additives were added. After the meat batter was compacted, it was filled into a 45-mm diameter edible casing. The stuffed sausages were then transferred to a smoker (oven) and subjected to drying (50–55 °C), smoking (65–70 °C), and blanching (80–85 °C). Before being packed, the sausages were quickly cooled.

As for sausages produced by P2, the cooled or frozen beef meat and fat were chopped using a cutter; salt, spices, and additives were then added. The meat batter was filled into edible casing with a diameter of 40 mm. Cold smoking (20–22 °C) was applied for 4–7 days. Information on drying and blanching steps was not provided by producer P2.

Regarding sausages produced by P3, the cooled or frozen beef meat and fat were ground in a cutter where the spices and additives were also added. After the meat batter was compacted, it was placed in an edible casing with a diameter of 24 mm. Like samples of P1, the stuffed sausages were dried (50–55 °C), smoked (65–70 °C), and blanched (80–85 °C). The sausages were then quickly cooled before being packed.

No other information was provided by the three producers.

Each sausage sample was collected aseptically, transported to the laboratory under refrigeration (+4 °C), and analyzed within two days from arrival.

**Fig. 1:**
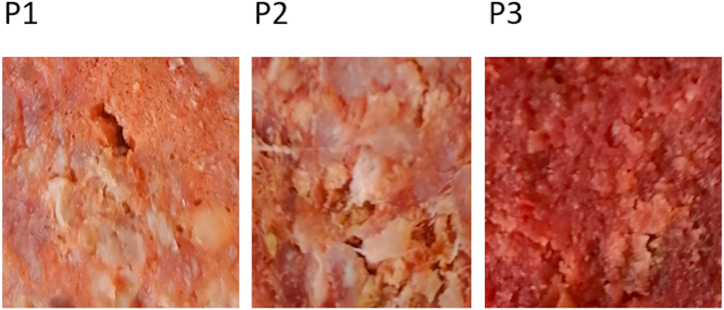
Examples of cross sections of smoked sausages of bovine meat. P1, producer 1; P2, producer 2; P3, producer 3.

Examples of cross sections of the smoked sausages of bovine meat herein studied are showed in Fig. 1.

### 2.2 Physico-chemical analyses

#### 2.2.1 Moisture content

For the determination of moisture content, 5 g of samples were dried in a hot air oven at a temperature of 105 °C [22] until a constant mass weight was achieved. The percentage moisture was calculated from the loss in mass of the test portion. Duplicate measurements were performed for each sample, and the results expressed as mean ± standard deviation.

#### 2.2.2 Fat content

The MKC ISO 1443:2021 method was used for the determination of the fat content in the samples. The method consists of boiling of a test portion with hydrochloric acid to free the occluded and bound lipid fractions, filtrating of the resulting mass, drying, and extracting, with petroleum ether, of the fat retained on the filter. Duplicate measurements were performed for each sample, and the results expressed as mean ± standard deviation.

#### 2.2.3 Protein content

Protein content was determined by measuring nitrogen (N × 6.25) with the Kjeldahl method (AOAC 981.10). Duplicate measurements were performed for each sample, and the results expressed as mean ± standard deviation.

#### 2.2.4 Chloride content

Total chloride content was determined by the method IS0 1841-1:1996 using Volhard procedure. The value was expressed as sodium chloride percentage by mass. Duplicate measurements were performed for each sample, and the results expressed as mean ± standard deviation.

#### 2.2.5 pH

The pH was assessed by directly inserting a HI2031 solid electrode (Hanna Instruments, Padova, Italy) into the center of the sausage. The measurements were preceded by a two-point calibration of the instrument with standard buffer solutions (pH 4 and 7). Duplicate measurements were performed for each sample, and the results expressed as mean ± standard deviation.

#### 2.2.6 Water activity

An AwTherm apparatus (Rotronic, Bassersdorf, Switzerland) was used to measure the water activity (a_w_) of the samples.

Duplicate measurements were performed for each sample, and the results expressed as mean ± standard deviation.

### 2.3 Morpho-textural analyses

Color was evaluated as already described by Osimani et al. [10]. In more detail, the measurements (10° viewing angle) were performed on 2 cm thick slices according to the CIE L*a*b* system. Lightness, redness (+)/greenness (−) color attribute, and yellowness (+)/blueness (−) color attribute were determined through *L*, a*, b* parameters, respectively. Moreover, hue (*h°*) and chroma (*C*) values were evaluated [23]. All the measurements were performed in triplicate for each sample, and the results expressed as mean ± standard deviation.

Texture profile analysis (TPA) was assessed as already described by Osimani et al. [10] using a CT3-4500 texture analyzer (Brookfield Engineering Laboratories Inc., Middleboro MA, USA) equipped with a 36 mm diameter cylindrical probe (mod. TA-AACC36), and a 4500 g load cell. For each sample, three independent measurements were carried out, and the results expressed as mean ± standard deviation.

### 2.4 Microbiological analyses

The analyzed smoked sausages were subjected to viable plate counting for the enumeration of different microbial groups. To this end, 10 g of each sample were homogenized in 90 mL of 0.1 % sterile peptone water for 3 min at 260 rpm with a Stomacher (400 Circulator, International PBI, Milan, Italy). Thus, decimal serial dilutions were prepared, and 1 mL-aliquots were inoculated in duplicate into selective media for the enumeration of the following microbial groups: (i) presumptive lactobacilli on De Man, Rogosa, and Sharpe (MRS) agar (VWR, Leuven, Belgium), supplemented with 250 mg L^−1^ of cycloheximide, with incubation at 30 °C for 48 h; (ii) coagulase-negative cocci (CNC) on Mannitol Salt Agar (MSA) (Liofilchem, Roseto degli Abruzzi, Italy) with incubation at 37 °C for 24–48 h; (iii) yeasts and molds on Rose Bengal (RB) agar (VWR) with incubation at 25 °C for 48–72 h; iv) Enterobacteriaceae in Violet Red Bile Glucose Agar (VWR) with incubation at 37 °C for 24 h; v) *Escherichia coli* in Chromogenic Coliform Agar (CCA) medium (VWR) incubated at 37 °C for 24 h; vi) coagulase-positive staphylococci on Baird Parker agar base (BP) medium (VWR) supplemented with Egg Yolk Tellurite Emulsion (VWR) incubated at 37 °C for 24 h. After incubation, only plates containing between 30 and 300 colonies were considered for viable counting.

The presence/absence of *L. monocytogenes* and *Salmonella* spp. was assessed as already described by Haouet et al. [24] through the enzyme-linked fluorescent assay (ELFA) method [24]. This automated immunoassay detects specific antigens using enzyme-labeled antibodies, producing fluorescence as a measurable signal. The procedure followed the AFNOR BIO 12/11–03/04 and AFNOR BIO 12/16–09/05 standard methods, respectively, for these pathogens. The analysis was performed in duplicate for each sample.

### 2.5 Assessment of pro-technological microorganisms

#### 2.5.1 Isolation

Colonies from microbial viable counts grown on MRS agar and MSA agar were randomly selected starting from the plates with the highest dilution factor. The colonies were cultivated to obtain pure cultures using streak dilution under the same growth conditions. A total of 30 bacterial isolates were collected, including 19 presumptive lactobacilli and 11 presumptive coagulase-negative cocci. In order to ensure the proper conservation of the purified isolates, a solution containing glycerol as cryoprotective was utilized for each culture. In more detail, a sterile solution containing glycerol and water was mixed (1:1) with MRS broth and brain heart infusion (BHI) broth (VWR) for presumptive lactobacilli or coagulase-negative cocci, respectively. Finally, microbial suspensions were aliquoted in sterile tubes, and then stored at −80 °C until use.

#### 2.5.2 DNA extraction and amplification

Thawed microbial isolates were sub-cultured twice and subjected to DNA extraction and amplification according to Osimani et al. [4]. For amplification, the following cycling program was used: initial denaturation at 95 °C for 3 min, followed by 35 cycles of denaturation at 95 °C for 1 min, annealing at 55 °C for 1 min and extension at 72 °C for 2 min. Amplicons were sequenced by Genewiz (Takaley, UK), and sequences were then compared with those of type strains from the GenBank DNA database (https://www.ncbi.nlm.nih.gov/). Sequences were finally submitted to the GenBank DNA database, and accession numbers were obtained.

#### 2.5.3 Occurrence of the hdcA gene

DNA extracts obtained from microbial isolates were tested for the presence of the *hdcA* gene. The PCR reactions were carried out using the primer pair Hdc1 (5′-TTGACCGTATCTCAGTGAGTCCAT-3′) and Hdc2 (5′- ACGGTCATACGAAACAATACCATC-3′) to amplify a fragment of 174 bp of the *hdcA* gene [25]. The PCR was carried out through a MyCycler Thermal Cycler (BioRad Laboratories) in a final volume of 25 μL for each reaction tube with the following cycling conditions: initial denaturation step at 95 °C for 3 min, 40 amplification cycles (95 °C for 20 s, 58 °C for 30 s, 72 °C for 20 s), and a final elongation step at 72 °C for 10 min. The results were checked by electrophoretic run on agarose gel. The analysis was carried out together with a blank, and the positive strain *Lactobacillus parabuchneri* DSM 5987.

#### 2.5.4 Antimicrobial activity

The method previously described by Cardinali et al. [26] was used. In more detail, the medium Brain Heart Infusion (BHI) (VWR) soft agar (0.75 % agar) was inoculated with the target microorganism *L. innocua* (2 %, v/v). After pouring 20 mL of the inoculation media into a Petri dish, wells of ∼50 μL were generated. After collecting an aliquot (500 μL) of the 24 h grown lactic acid bacteria culture, the remaining broth cultures were centrifuged (1,610×*g* for 10 min). Then, the supernatant was added with 0.1 N NaOH to reach pH 7 in order to neutralize the organic acids produced during bacterial growth. An aliquot (500 μL) of the neutralized supernatant was collected, and finally filtrated with a 0.22 μm pore size filter. Thus, for each isolate, the BHI soft agar Petri dishes were inoculated with: (i) 50 μL of the sub-cultured suspension; (ii) 50 μL of the neutralized suspension; and (iii) 50 μL of the filtered suspension. The Petri dishes were incubated at 37 °C for 24 h and the antimicrobial activity (presence of zones of inhibition) was confirmed as already described by Cardinali et al. [26].

#### 2.5.5 Production of EPS

The selected isolates were assessed for the production of EPS according to the method previously described by Hilbig et al. [27] with few adjustments. First, the isolates were extracted form cryo-protective solutions and sub-cultured twice for 48 h at 30 °C. Then, 5 μL-aliquots of each bacterial culture were added to the following solid media: MRS agar with sucrose (80 g L^−1^); MRS agar with yeast extract (VWR Chemicals) (10 g L^−1^), meat extract (VWR Chemicals) (10 g L^−1^), lactose (Carlo Erba Reagents, Cornaredo, Italy) (20 g L^−1^), and galactose (VWR Chemicals) (20 g L^−1^) [28]. All the agar plates were incubated for 48 h at 30 °C.

Positive colonies showed a ropy consistency or a mucoid aspect. For each isolate, duplicate analyses were performed.

#### 2.5.6 Enzymatic activities

The assessment of the enzymatic activities was performed as described by Linares-Morales et al. [29] with some modifications. Prior to the test, the isolates were sub-cultured twice on the same medium previously used for the isolation.

Esterase activity was determined on Tween 80 agar [29], whereas skim milk agar [29] and tributyrin agar [29] were used for the evaluation of proteolytic and lipolytic activity, respectively. Aliquots (5 μL) of each bacterial culture were spotted onto agar plates and incubated at 30 °C for 48 h. The presence of an opaque precipitate (esterase activity) or a clear halo (proteolytic and lipolytic activity) around the inoculum indicated a positive result, with intensity levels denoted as + (1 mm), ++ (1–2 mm), and +++ (3 mm) [30].

#### 2.5.7 Acidification performance

The tested lactic acid bacteria cultivated on MRS broth were centrifuged at 1,610×*g* for 5 min using a Rotofix 32A centrifuge (Hettich, Milano, Italy) and the pellets washed with sterile physiological solution (0.9 % w v^-1^) prior to resuspension in the same diluent. The optical density (OD) at 600 nm of the bacterial cells’ concentration was determined through a spectrophotometer (Shimadzu UV-1800, Shimadzu Corporation, Kyoto, Japan). The tested lactic acid bacteria were inoculated to 8 log cfu mL^−1^ concentration in 10 mL of the following liquid growth media: Medium 1) MRS broth (VWR); Medium 2) MRS broth (VWR) supplemented with 150 mg kg^−1^ of sodium nitrite (Tec-Al, Traversetolo, Italy); Medium 3) MRS broth (VWR) supplemented with 150 mg kg^−1^ of potassium nitrate (Tec-Al); Medium 4) MRS broth (VWR) added with 3 % (w v^−1^) of sodium chloride (Italkali, Palermo, Italy); Medium 5) MRS broth (VWR) supplemented with 150 mg kg^−1^ of sodium nitrite (Tec-Al), 150 mg kg^−1^ of potassium nitrate (Tec-Al), and 3 % (w v^−1^) of sodium chloride (Italkali). The pH values were assessed before inoculation (t0) and after incubation for 4 and 24 h at 30 °C. Uninoculated broth media were used as control.

#### 2.5.8 Nitrate reductase activity

The nitrate reductase activity was assessed following the procedure outlined by Miralles et al. [31] with some minor adjustments proposed by Jeong et al. [32]. Colonies grown on TSA were transferred to YT agar (tryptone 1.0 %, yeast extract 0.5 %, pH 7.0) supplemented with 0.1 % KNO_3_. Following a 20-h incubation at 30 °C, the plates were flooded with a mixture of solution NIT1 (0.8 g of sulfanilic acid in 100 mL of 5 N acetic acid) and NIT2 (0.6 g of N,N-dimethyl-1-naphthylamine in 100 mL of 5 N acetic acid) for nitrite detection. The presence of red halos surrounding colonies indicated nitrate reductase activity.

### 2.6 Statistical analysis

Statistical differences among samples were assessed using one-way analysis of variance (ANOVA) followed by Tukey-Kramer's Honest Significant Difference (HSD) test, with a significance level of 0.05. Moreover, a Principal Component Analysis (PCA) was performed on data from compositional, physicochemical, and morpho-textural analysis.

The tests were conducted using JMP software, version 11.0.0 (SAS Institute Inc., Cary, NC).

## 3 Results

### 3.1 Physico-chemical and morpho-textural characterization

The results of proximate composition of the smoked sausages of bovine meat are reported in Table 1.

**Table 1:** Proximate composition of smoked sausages of bovine meat.

Producer	Moisture (%)	Protein (%)	Fat (%)	NaCl (%)
1	48.61 ± 1.05^A^	26.85 ± 0.06^B^	18.00 ± 0.07^B^	4.58 ± 0.05^B^
2	29.37 ± 0.06^C^	29.41 ± 0.20^A^	18.65 ± 0.10^B^	4.97 ± 0.03^A^
3	33.70 ± 0.44^B^	29.19 ± 0.10^A^	22.95 ± 0.13^A^	5.04 ± 0.02^A^

For each producer, values are expressed as mean ± standard deviation of pooled samples from 3 production batches. For each producer, different letters in the same column indicate significant differences according to the Tukey–Kramer's (HSD) test (=0.05).

Regarding moisture content, samples of producer 1 showed the highest average value (48.61 %), whereas samples of producer 2 showed the lowest (29.37 %). As for protein content, samples from producer 1 exhibited a significantly lower average value (26.85 %) compared to the other samples (*p* < 0.05). Fat content was significantly higher (*p* < 0.05) in samples of producer 3 (22.95 %). Regarding salt (NaCl) content, samples from producer 1 exhibited the lowest average value (4.58 %).

The results of pH and a_w_ measurements are reported in Table 2.

**Table 2:** pH and water activity (a_w_) values of smoked sausages of bovine meat.

Producer	Batch	Sample code	pH	a_w_
1	1	P1S1	6.04 ± 0.03^a^	0.94 ± 0.00^b^
	2	P1S2	6.13 ± 0.06^a^	0.96 ± 0.01^a^
	3	P1S3	6.02 ± 0.07^a^	0.96 ± 0.00^a^
		Overall mean	6.06 ± 0.07^A^	0.95 ± 0.01^A^
2	1	P2S1	4.90 ± 0.02^a^	0.88 ± 0.01^a^
	2	P2S2	4.92 ± 0.01^a^	0.87 ± 0.00^a^
	3	P2S3	4.87 ± 0.01^a^	0.89 ± 0.00^a^
		Overall mean	4.90 ± 0.02^B^	0.88 ± 0.01^C^
3	1	P3S1	6.12 ± 0.02^a^	0.90 ± 0.00^a^
	2	P3S2	6.14 ± 0.02^a^	0.90 ± 0.01^a^
	3	P3S3	6.11 ± 0.02^a^	0.91 ± 0.01^a^
		Overall mean	6.12 ± 0.02^A^	0.90 ± 0.01^B^

For each producer, values are expressed as mean ± standard deviation.

For each producer, lowercase letters indicate significant differences among samples of the same producer, whereas capital letters indicate differences among overall means according to the Tukey–Kramer's (HSD) test (=0.05).

The average pH value of samples of producer 2 was significantly lower (4.90) compared to producer 1 and 3 (6.06 and 6.12, respectively) (*p* < 0.05). As for a_w_ values, statistically significant differences were observed among mean values (*p* < 0.05), with producer 1 showing the highest value (0.95) and producer 2 the lowest (0.88).

The results of morpho-textural evaluation of the analyzed smoked sausages are reported in Table 3.

**Table 3:** Morpho-textural characteristics of smoked sausages of bovine meat.

Producer	Batch	Sample code	Hardness (N)	Cohesiveness	Springiness
1	1	P1S1	1.97 ± 0.32^a^	0.87 ± 0.01^a^	1.80 ± 0.00^a^
	2	P1S2	2.17 ± 0.86^a^	0.89 ± 0.01^a^	1.85 ± 0.07^a^
	3	P1S3	2.00 ± 0.85^a^	0.84 ± 0.04^a^	1.80 ± 0.00^a^
		Overall mean	2.04 ± 0.57^B^	0.87 ± 0.03^B^	1.82 ± 0.04^A^
2	1	P2S1	39.61 ± 6.75^a^	0.80 ± 0.02^a^	1.75 ± 0.07^a^
	2	P2S2	40.88 ± 6.27^a^	0.77 ± 0.01^a^	1.70 ± 0.14^a^
	3	P2S3	42.97 ± 7.14^a^	0.81 ± 0.05^a^	1.75 ± 0.07^a^
		Overall mean	41.15 ± 5.43^A^	0.79 ± 0.03^C^	1.73 ± 0.08^B^
3	1	P3S1	3.51 ± 0.05^a^	0.91 ± 0.01^a^	1.85 ± 0.07^a^
	2	P3S2	3.62 ± 0.06^a^	0.92 ± 0.02^a^	1.90 ± 0.00^a^
	3	P3S3	3.61 ± 0.23^a^	0.91 ± 0.02^a^	1.80 ± 0.00^a^
		Overall mean	3.58 ± 0.12^B^	0.91 ± 0.02^A^	1.85 ± 0.05^A^

For each producer, values are expressed as mean ± standard deviation.

For each producer, lowercase letters indicate significant differences among samples of the same producer, whereas capital letters indicate differences among overall means according to the Tukey–Kramer's (HSD) test (=0.05).

Regarding hardness, the overall value of samples of producer 2 was significantly higher (41.15 N) compared to the other samples (*p* < 0.05). As for cohesiveness, the overall value of samples of producer 3 was the highest (0.91), whereas that of producer 2 was the lowest (0.79). Considering springiness, the overall value of samples of producer 2 was the lowest (1.73).

The results of the color evaluation of the analyzed bovine sausages are reported in Table 4.

**Table 4:** Color evaluation of smoked sausages of bovine meat.

Producer	Batch	Sample code	L*	a*	b*	*h°*	*C*
1	1	P1B1	45.33 ± 1.40^a^	16.84 ± 0.04^a^	15.64 ± 1.42^a^	42.81 ± 2.59^a^	23.00 ± 0.99^a^
	2	P1B2	46.01 ± 0.19^a^	16.43 ± 0.20^a^	15.87 ± 1.16^a^	43.96 ± 1.77^a^	22.85 ± 0.93^a^
	3	P1B3	43.02 ± 0.34^b^	17.07 ± 0.33^a^	15.17 ± 0.64^a^	41.62 ± 0.88^a^	22.84 ± 0.62^a^
		Overall mean	44.79 ± 1.54^A^	16.78 ± 0.34^B^	15.56 ± 1.02^B^	42.79 ± 1.92^A^	22.90 ± 0.75^B^
2	1	P2B1	44.24 ± 1.41^a^	16.29 ± 0.61^b^	10.26 ± 1.57^b^	32.07 ± 3.06^b^	19.27 ± 1.34^b^
	2	P2B2	45.97 ± 0.51^a^	17.87 ± 0.08^a^	17.32 ± 0.12^a^	44.10 ± 0.27^a^	24.89 ± 0.08^a^
	3	P2B3	45.48 ± 0.16^a^	16.83 ± 0.25^b^	9.57 ± 0.32^b^	29.61 ± 0.47^b^	19.36 ± 0.38^b^
		Overall mean	45.23 ± 1.08^A^	17.00 ± 0.77^B^	12.38 ± 3.80^C^	35.26 ± 6.89^B^	21.17 ± 2.87^B^
3	1	P3B1	31.28 ± 0.21^a^	21.21 ± 0.34^a^	21.13 ± 0.56^a^	44.89 ± 0.32^a^	29.93 ± 0.63^a^
	2	P3B2	31.41 ± 0.11^a^	19.69 ± 0.33^b^	18.44 ± 0.10^a^	43.12 ± 0.64^a^	26.98 ± 0.18^b^
	3	P3B3	30.89 ± 0.58^a^	19.03 ± 0.31^b^	19.69 ± 1.94^a^	45.89 ± 2.32^a^	27.39 ± 1.61^b^
		Overall mean	31.19 ± 0.39^B^	19.98 ± 1.01^A^	19.75 ± 1.54^A^	44.63 ± 1.72^A^	28.10 ± 1.64^A^

For each producer, values are expressed as mean ± standard deviation.

For each producer, lowercase letters indicate significant differences among samples of the same producer, whereas capital letters indicate differences among overall means according to the Tukey–Kramer's (HSD) test (=0.05).

L* value describes the lightness; a* value describes the redness/greenness; b* describes the blueness/yellowness; *h°* value describes the hue; *C* value describes the chroma.

The overall L* mean value of samples of producer 3 was significantly lower (31.19) than overall means of producer 1 and 2 (44.79 and 45.23, respectively) (*p* < 0.05). For red/green opponents (a*), the mean value of samples of producer 3 was significantly higher (19.98) than the overall means of producer 1 and producer 2 (16.78 and 17.00, respectively) (*p* < 0.05). As for the blue/yellow opponents (b*), the mean value of samples of producer 3 was the highest (19.75), whereas that of producer 2 was the lowest (12.38).

As for hue (*h°*), samples of producer 2 showed significantly lower average value (35.26) compared to the other samples (*p* < 0.05). The average chroma (*C*) level was the highest in samples of producer 3 (28.10).

**Fig. 2:**
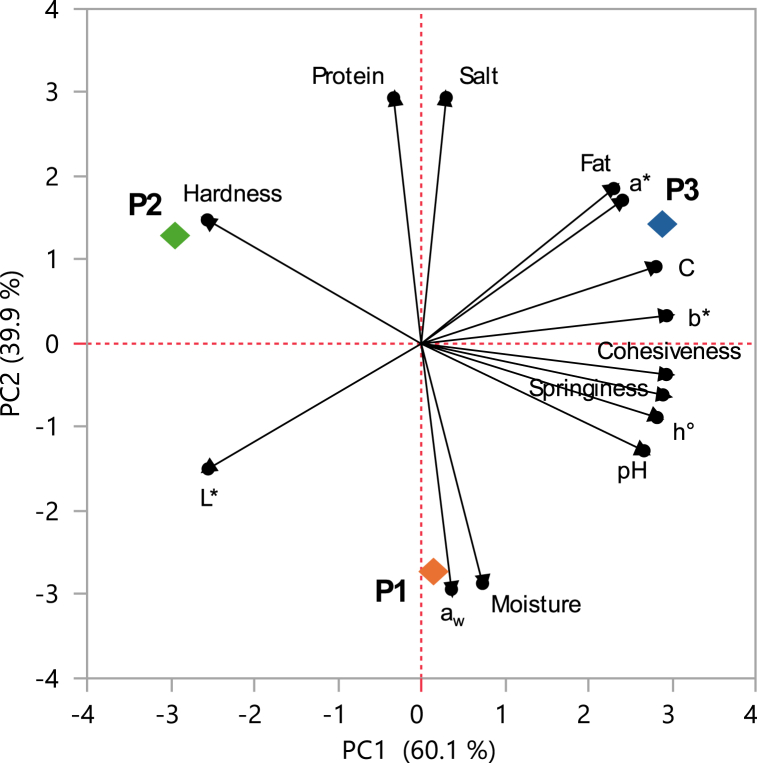
Principal Component Analysis (PCA) of sausages collected from producer 1 (P1), producer 2 (P2), and producer 3 (P3) based on compositional, physicochemical, and morpho-textural parameters.

Fig. 2 shows the results of the PCA based on the compositional, physicochemical, and morpho-textural parameters of the analyzed sausages. Two principal components were identified from the correlation matrix. PC1 and PC2 accounted for 60.1 and 39.9 % of the total variation, respectively. PC1 neatly separated the sausages collected from the three different producers (P1, P2, P3); PC2 separated P1 sausages from those collected from P2 and P3. Moisture and a_w_ were negatively correlated with protein and salt content; fat content resulted negatively correlated with lightness (L*).

### 3.2 Microbiological analyses

The results of viable counts performed on the smoked sausages of bovine meat herein analyzed are reported in Table 5.

**Table 5:** Viable counts detected in the analyzed smoked sausages of bovine meat.

Producer	Batch	Sample code	Presumptive lactobacilli	Coagulase-negative cocci	Yeasts	Molds	Enterobacteriaceae	*Escherichia coli*	Coagulase-positive staphylococci
1	1	P1S1	4.41 ± 0.04^a^	7.69 ± 0.13^a^	6.45 ± 0.01^a^	<1	<1	<1	<1
	2	P1S2	3.94 ± 0.34^a^	7.44 ± 0.02^a^	6.04 ± 0.03^b^	<1	<1	<1	<1
	3	P1S3	5.16 ± 0.52^a^	7.29 ± 0.08^a^	5.97 ± 0.09^b^	<1	<1	<1	<1
		Overall mean	4.50 ± 0.62^B^	7.47 ± 0.20^A^	6.15 ± 0.24^A^	<1	<1	<1	<1
2	1	P2S1	8.93 ± 0.08^a^	5.94 ± 0.00^a^	5.11 ± 0.08^a^	<1	<1	<1	<1
	2	P2S2	8.41 ± 0.04^b^	5.86 ± 0.06^a^	4.56 ± 0.00^b^	<1	<1	<1	<1
	3	P2S3	8.60 ± 0.08^b^	5.06 ± 0.02^b^	4.62 ± 0.07^b^	<1	<1	<1	<1
		Overall mean	8.65 ± 0.24^A^	5.62 ± 0.43^B^	4.76 ± 0.28^B^	<1	<1	<1	<1
3	1	P3S1	5.34 ± 0.06^a^	5.90 ± 0.01^a^	6.48 ± 1.27^a^	<1	<1	<1	<1
	2	P3S2	5.41 ± 0.01^a^	3.95 ± 0.02^c^	5.04 ± 0.09^a^	<1	<1	<1	<1
	3	P3S3	4.11 ± 0.05^b^	4.66 ± 0.05^b^	5.94 ± 0.05^a^	<1	<1	<1	<1
		Overall mean	4.95 ± 0.65^B^	4.84 ± 0.88^B^	5.82 ± 0.86^A^	<1	<1	<1	<1

For each producer, values are expressed as of log cfu g^−1^ mean ± standard deviation.

For each producer, lowercase letters indicate significant differences among samples of the same producer, whereas capital letters indicate differences among overall means according to the Tukey–Kramer's (HSD) test (=0.05).

Regarding presumptive lactic acid bacteria, the overall mean count of samples of producer 2 was significantly higher (8.65 log cfu g^−1^) compared to producer 1 and 3 (4.50 and 4.95 log cfu g^−1^, respectively) (*p* < 0.05). As for coagulase-negative cocci, the overall mean count of samples of producer 1 was the highest (7.47 log cfu g^−1^). Considering yeasts, the overall mean count of samples of producer 2 was the lowest (4.76 log cfu g^−1^). The counts of molds, Enterobacteriaceae, *E. coli*, and coagulase-positive staphylococci were <1 log cfu g^−1^ in all the samples.

In all the samples (25 g of each sausage), the absence of *L. monocytogenes* or *Salmonella* spp. was observed.

### 3.3 Isolation and characterization of lactic acid bacteria and coagulase-negative cocci

#### 3.3.1 Lactic acid bacteria

The BLAST search enabled the unambiguous identification of one *Weissella viridescens* and 18 *Latilactobacillus sakei* (basionym *Lactobacillus sakei*) isolates.

The results of identification of the isolates are reported in Table 6 (panel a).

**Table 6:** Identification of lactic acid bacteria (panel a) and coagulase-negative cocci (panel b) isolated from the analyzed smoked sausages of bovine meat.

Panel a			
Isolate code	Closest relative	% Identity^a^	Accession number^b^
S3	*Weissella viridescens*	99.66 %	NR_040813
S7	*Latilactobacillus sakei*	98.57 %	NR_113821
S8	*Latilactobacillus sakei*	99.25 %	NR_113821
S9	*Latilactobacillus sakei*	99.91 %	NR_113821
S10	*Latilactobacillus sakei*	98.57 %	NR_113821
S11	*Latilactobacillus sakei*	98.81 %	NR_113821
S12	*Latilactobacillus sakei*	99.17 %	NR_113821
S13	*Latilactobacillus sakei*	99.69 %	NR_113821
S14	*Latilactobacillus sakei*	99.64 %	NR_113821
S15	*Latilactobacillus sakei*	99.45 %	NR_113821
S16	*Latilactobacillus sakei*	99.09 %	NR_113821
S17	*Latilactobacillus sakei*	98.93 %	NR_113821
S18	*Latilactobacillus sakei*	98.18 %	NR_113821
S19	*Latilactobacillus sakei*	98.00 %	NR_113821
S23	*Latilactobacillus sakei*	99.63 %	NR_113821
S24	*Latilactobacillus sakei*	98.09 %	NR_113821
S25	*Latilactobacillus sakei*	98.40 %	NR_113821
S26	*Latilactobacillus sakei*	98.43 %	NR_113821
S27	*Latilactobacillus sakei*	98.35 %	NR_113821
Panel b			
Isolate code	Closest relative	% Identity^a^	Accession number^b^
S1	*Staphylococcus equorum*	98.98 %	NR_027520
S2	*Staphylococcus equorum*	99.63 %	NR_027520
S4	*Staphylococcus equorum*	99.28 %	NR_027520
S5	*Staphylococcus casei*	100.00 %	NR_037053
S6	*Staphylococcus equorum* subsp. *linens*	99.24 %	NR_041926
S20	*Staphylococcus equorum*	98.88 %	NR_027520
S21	*Staphylococcus casei*	99.74 %	NR_037053
S22	*Staphylococcus equorum*	99.67 %	NR_027520
S28	*Staphylococcus casei*	99.83 %	NR_037053
S29	*Staphylococcus casei*	99.83 %	NR_037053
S30	*Staphylococcus equorum*	98.36 %	NR_027520

a Percentage of identical nucleotides in the sequence obtained from the bacterial isolates and the sequence of the closest relative found in the GenBank database.

b Accession number of the sequence of the closest relative found by BLAST search.

The results of antimicrobial activity, esterase activity, lipase activity, protease activity, EPS production, and occurrence of the *hdcA* gene in the studied isolates are reported in Table 7.

**Table 7:** Characterization of the lactic acid bacteria isolated from the analyzed smoked sausages of bovine meat.

Isolate code	Closest relative	Antimicrobial activity	Esterase activity	Lipase activity	Protease activity	EPS production		*hdcA* gene
						Sucrose-dependent	Sucrose-independent	
S3	*Weissella viridescens*	–	+	–	++	–	–	–
S7	*Latilactobacillus sakei*	–	–	–	++	M	–	–
S8	*Latilactobacillus sakei*	–	–	–	+	–	–	–
S9	*Latilactobacillus sakei*	–	–	–	++	–	–	–
S10	*Latilactobacillus sakei*	–	–	–	+	–	–	–
S11	*Latilactobacillus sakei*	–	–	–	+	–	–	–
S12	*Latilactobacillus sakei*	–	–	–	+	–	–	–
S13	*Latilactobacillus sakei*	–	–	–	++	M	–	–
S14	*Latilactobacillus sakei*	–	–	–	+	–	–	–
S15	*Latilactobacillus sakei*	–	–	–	+	–	–	–
S16	*Latilactobacillus sakei*	–	–	–	+	–	–	–
S17	*Latilactobacillus sakei*	–	–	–	+	–	–	–
S18	*Latilactobacillus sakei*	–	–	–	+	–	–	–
S19	*Latilactobacillus sakei*	–	–	–	+	–	–	–
S23	*Latilactobacillus sakei*	–	–	–	+	–	–	–
S24	*Latilactobacillus sakei*	–	–	–	+	–	–	–
S25	*Latilactobacillus sakei*	–	–	–	++	–	–	–
S26	*Latilactobacillus sakei*	–	–	–	+	–	–	–
S27	*Latilactobacillus sakei*	–	–	–	++	M	–	–

-, negative; +, positive colonies; M, mucoid appearance.

Regarding the antimicrobial activity, no isolate was able to inhibit the growth of *L. innocua*.

As for the tested enzymatic activities, no isolate showed a positive reaction for lipase, whereas *W. viridiscens* showed a positive esterase activity. All the isolates showed protease activity, with isolates S3, S7, S9, S13, S25, and S27 being those with the strongest reaction.

Three out of the 19 lactic acid bacteria showed visible glossy and slimy look (mucoid colonies) in the MRS agar added with sucrose, thus suggesting the production of sucrose-dependent EPS. No mucoid colonies or colonies with ropy consistency were observed in MRS added with lactose and galactose, thus suggesting the absence of sucrose-independent EPS.

No isolate showed the presence of the *hdcA* gene.

**Fig. 3:**
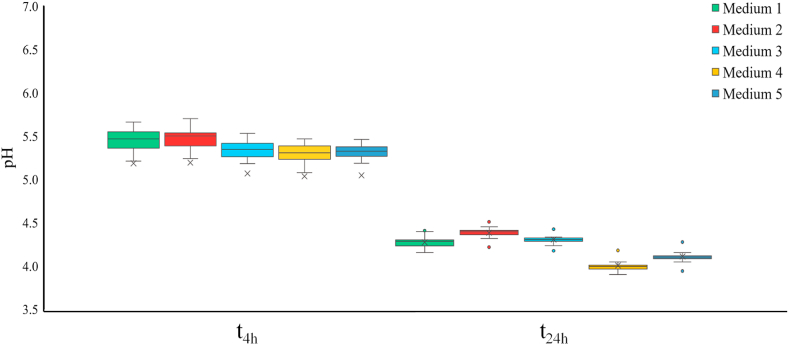
Box plots summarizing the results of acidification performance of the *Latilactobacillus sakei* isolates in synthetic media after 4 and 24 h. For each box, the bottom whisker marks the minimum value, the bottom of the box marks the location of first quartile, the line within the box refers to the median value, the top of the box marks the location of the third quartile, the top whisker marks the maximum value in the data set, the “X” symbol marks the average value, and circles indicate the outliers. Medium 1, MRS broth; Medium 2, MRS broth +150 mg kg-1 E250; Medium 3, MRS broth +150 mg kg-1 E252; Medium 4, MRS broth +3 % NaCl; Medium 5, MRS broth +150 mg kg-1 E250 + 150 mg kg-1 E252 + 3 % NaCl.

The results of the acidification performance of the 18 *Lat. sakei* isolates are reported in Fig. 3.

In more detail, pH values measured in the uninoculated growth media were 6.28, 6.41, 6.37, 6.17, and 6.17 in Medium 1, Medium 2, Medium 3, Medium 4, and Medium 5, respectively.

Regarding acidification in Medium 1, isolate S13 showed the highest pH after 24 h (4.43), while the isolates S7 and S26 reached the lowest pH values (4.19) after 24 h.

As for acidification in Medium 2, the isolate S12 showed the highest pH (4.52) after 24 h, while the medium inoculated with the isolate S26 reached the lowest pH (4.25) after 24 h.

Regarding Medium 3, the isolates S12 and S13 showed the highest pH (4.46 and 4.44, respectively) after 24 h, while the medium inoculated with the isolates S7, S26, S27 showed the lowest pH value (4.20) after 24 h.

In Medium 4, the isolates S12 and S13 showed the highest pH value after 24 h (4.21). The growth medium inoculated with the isolates S7, S26, and S27 reached the lowest pH (3.95) after 24 h.

Regarding Medium 5, the isolates S12 and S13 showed the highest pH values after 24 h (4.30), while the medium inoculated with the isolates S7, S26, and S27 reached the lowest pH (4.00) after 24 h.

The data overall collected showed a low variability of the results among the isolates.

The *W. viridiscens* isolate was not tested for acidification performance.

In the uninoculated broth media no changes in pH were observed after 4 or 24 h.

#### 3.3.2 Coagulase-negative cocci

Seven *Staphylococcus equorum* and 4 *Staphylococcus casei* (basionym *Staphylococcus succinus* subsp. *casei*) isolates were identified (Table 6, panel b).

Table 8 shows the results of antimicrobial activity, nitrate reductase, esterase activity, lipase activity, protease activity, EPS production, and occurrence of the *hdcA* gene in the studied coagulase-negative cocci.

**Table 8:** Characterization of the coagulase-negative cocci isolated from the analyzed smoked sausages of bovine meat.

Isolate code	Closest relative	Antimicrobial activity	Esterase activity	Lipase activity	Protease activity	EPS production		*hdcA* gene	Nitrate reductase
						Sucrose-dependent	Sucrose-independent		
S1	*Staphylococcus equorum*	–	–	+	+	–	–	–	+
S2	*Staphylococcus equorum*	–	–	+	+	–	–	–	–
S4	*Staphylococcus equorum*	–	–	–	+	–	–	–	+
S5	*Staphylococcus casei*	–	–	–	++	–	–	–	–
S6	*Staphylococcus equorum* subsp*. linens*	–	–	–	+	–	–	–	+
S20	*Staphylococcus equorum*	–	–	–	+	–	–	–	+
S21	*Staphylococcus casei*	–	–	++	+	M	–	–	–
S22	*Staphylococcus equorum*	–	–	–	+	–	–	–	+
S28	*Staphylococcus casei*	–	–	++	+	M	–	–	–
S29	*Staphylococcus casei*	–	–	–	+	–	–	–	–
S30	*Staphylococcus equorum*	–	–	–	+	–	–	–	+

-, negative; +, positive colonies; M, mucoid appearance.

No isolate was able to inhibit the growth of *L. innocua*.

Regarding the tested enzymatic activities, no isolate showed a positive reaction for esterase.

Regarding lipase, 4 out of the 11 isolates were positive for this enzymatic activity, with isolates S21 and S28 being those with the strongest reaction. All the isolates showed protease activity, with isolate S5 (*S. casei*) being the one with the strongest reaction.

Two out of the 11 coagulase-negative cocci showed visible glossy and slimy look (mucoid colonies) in the MRS agar added with sucrose; whereas no mucoid colonies or colonies with ropy consistency were observed in MRS added with lactose and galactose.

No isolate showed the presence of the *hdcA* gene.

Regarding nitrate reductase activity, 6 out of the 7 *S. equorum* isolates were positive for this enzymatic activity, whereas no isolate of *S. casei* showed a positive reaction.

## 4 Discussion

In smoked sausages, whether cold- or hot-smoked, it is supposed that the temperature reached during smoking is able to strongly inhibit the autochthonous microbiota occurring in the raw material. However, there is evidence that such processed sausages are far from being free of viable microorganisms [12,14,17,[33], [34], [35]]. Notably, although technically unfermented, the examined smoked sausages displayed physicochemical and microbiological characteristics comparable to fermented ones. Therefore, the findings of this study are discussed in relation to those documented in the scientific literature concerning fermented or smoked sausages.

As far as the proximate composition is concerned, the detected values for moisture, protein, and fat were similar to those observed by Škaljac et al. [7] in dry fermented beef sausage (Sjenički sudžuk) from Serbia. In these latter product moisture content ranged from 32 to 35 %, protein from 29 to 32 %, and fat from 24 to 26 % [7]. Of note, the proximate composition of sausages is strongly affected by the proportion of meat and fat used to produce the meat batter, as well as by the ripening conditions that can influence the moisture of the end product.

As reported by Belleggia et al. [12], the muscle and fat amount also affect the color of the sausage. In the CIE L*a*b* system, the a* parameter is used to denote the green–red opponent colors (<0 toward green and >0 toward red). In the samples herein analyzed the lowest a* average value was measured in samples with the lowest protein content. This finding is in agreement with the study carried out by Belleggia et al. [12] regarding Polish smoked sausages. Concerning the b* parameter, it represents the blue–yellow opponent colors (<0 toward blue and >0 toward yellow). In the samples of smoked sausages studied in the present research, those containing the highest amount of fat (producer 3) showed the highest yellow levels. This result is in accordance with the studies on sausages (fermented or non-fermented) carried out by Osimani et al. [10] and Belleggia et al. [12]. Indeed, the increased yellow color in sausages containing high percentages of lard, as a result of possible fat rancidity due to lipid oxidation, was observed by the same authors [10,12]. In the present study, the results of color analysis showed L* values that were similar to those observed by Škaljac et al. [7] in Sjenički sudžuk sausages. The color of sausages can strongly be influenced by several parameters that include moisture content, salt addition, added spices, pH value, meat type, and microbial communities (e.g., coagulase-negative cocci) [7], as well as process conditions. The lowest hue (*h°*) average value of samples of producer 2 likely reflects the effect of fermentation followed by low-temperature smoking. These combined processes could affect the final color of the meat batter. Regarding chroma (*C*) values, the higher color saturation in samples from producer 3 is consistent with the highest b* parameter values, indicating a shift towards red.

Regarding the morpho-textural traits, the highest hardness average value was observed in samples of producer 2. Indeed, samples from producer 2 were those subjected to fermentation and showing the lowest moisture, thus likely explaining the highest level of hardness.

The cohesiveness value serves as a measure to assess the extent to which a tested sample maintains its integrity when subjected to a second deformation in comparison to its resistance to the initial deformation. As reported by Baer and Dilger [36] and Cáceres et al. [37], the different cohesiveness values observed in the sausage samples under study can be the result of the fat content. The samples of producer 3, showing the highest amount of fat, were also those showing the highest cohesiveness (as also evidenced by PCA analysis).

As for springiness, this is an index of elastic recovery, showing how quickly a deformed sample returns to its original state once the force causing the deformation is removed. In this study, the lowest springiness was observed in smoked sausages of producer 2 that were also those with the highest hardness and lowest moisture content. Thus, suggesting a strong relationship among these three parameters (as also evidenced by PCA analysis).

The a_w_ of a food represents the ratio of the vapor pressure of the food in a state of equilibrium with the surrounding air medium to the vapor pressure of distilled water under the same conditions. Microorganisms can solely transport nutrients in and out of cells through the cell wall; hence, the nutrients must be in soluble form to penetrate the cell. In the food matrix, a portion of the overall water content is tightly bound to specific sites and does not function as a solvent. Noteworthy is that the correlation between water content and a_w_ does not exhibit a linear pattern across the entire range of a_w_ values and is instead characterized by non-linear mathematical equations, as described by sorption isotherms. These equations are contingent upon the interaction between food components and water. Notwithstanding, in the present study, the a_w_ levels of the analyzed sausages were in accordance with their moisture content. The a_w_ values of samples of producer 2 were in accordance with those reported by Ikonić et al. [38] for the fermented sausages of bovine meat Sjenički sudžuk produced in the town of Sjenica (Serbia) accounting at 0.85 after 23 days of ripening. However, very diverse a_w_ values have already been reported in the scientific literature for this type of fermented sausage (e.g., sucuk salami) based on the diverse production processes [39], thus explaining the high a_w_ values detected in samples of producer 1 and 3. The a_w_ results herein collected are consistent with those reported by Belleggia et al. [40] for the Portuguese smoked sausage cacholeira.

Concerning pH, producer 2 showed low values, while pH values of approximately 6 were observed in samples of producer 1 and 3. Interestingly, samples of producer 2 showed the highest lactic acid bacteria counts and the lowest yeast counts. Conversely, in samples of producer 1 and 3, the lowest counts of lactic acid bacteria were observed, together with the highest yest counts, thus likely explaining the differences among pH values detected in the analyzed smoked sausages. Of note, yeasts are able to metabolize organic acids produced by lactic acid bacteria, thus leading to an increase in pH values of the end product [38,39].

The PCA performed on the compositional, physicochemical, and morpho-textural data clearly separated the three producers. This result is particularly of interest since it clearly highlights that, although samples were characterized by different compositional, physicochemical, and morpho-textural traits, the viable microorganisms occurring in the meat batter, and dominating during processing (including fermentation and/or smoking), were represented by the same key species adapted to the meat environment. However, since no starter cultures were applied in the production process of the smoked sausages under investigation, it is challenging to precisely attribute variations in bacterial loads. In fact, the microbiology of derived meat products, like smoked sausages, depends on the quality of the raw meat and the applied processing conditions [41]. In the Republic of North Macedonia, sausage production is a traditional craft, maintained through the careful preservation of specific recipes and methods by local artisans and home-based producers [42,43]. Therefore, the observed differences in smoked sausages from different producers might result from variations in the technological conditions applied during their manufacturing.

Producer 2 showed counts of lactic acid bacteria that were in accordance with those detected by Ercoşkun & Özkal [39] in sucuk salami produced in Izmir (Türkiye) that showed count of ∼7 log cfu g^−1^. Since samples of producer 1 and producer 3 were not subjected to fermentation, the lower counts of lactic acid bacteria were not unexpected.

In meat-based products, lactic acid bacteria can exert a double effect. On the one hand, they could be the causative agent of meat spoilage (e.g., in raw meat or in cooked products), on the other hand, they could contribute in producing organic acids (mainly lactic and acetic acids) and other active compounds that strongly affect the quality and safety of fermented foods. Moreover, organic acids synthesized by lactic acid bacteria help in inhibiting the multiplication of undesired microorganisms (e.g., Enterobacteriaceae, spoilage bacteria, etc.) naturally occurring in the raw materials [44]. Additionally, lactic acid bacteria metabolism helps in stabilizing the color of meat and developing the texture, such as the formation of the typical gel-like consistency [45]. As reported by Xia et al. [46], lactic acid bacteria can also enable the breakdown of lipids, proteins, and carbohydrates, leading to the generation of small molecules, such as peptides, or volatile compounds. Intriguingly, lactic acid bacteria can regulate the lipid oxidation process through the expression of antioxidant enzyme genes and can produce lipase to help the degradation of triglycerides, with a positive effect on the quality and flavor of sausages [46]. Other beneficial molecules may also be produced by lactic acid bacteria, including antimicrobial compounds (e.g., bacteriocins), and EPS, thus effectively improving the safety and texture of the sausages.

As recently reviewed by Stegmayer et al. [44], the adoption of starter or adjunct lactic acid bacteria cultures by the meat industry plays a crucial role in enhancing the quality of fermented sausages or even stabilizing raw meat, thereby standardizing technological processes and ensuring product safety. Consequently, in the present study, lactic acid bacteria were isolated and subsequently characterized for various pro-technological traits.

The isolation campaign allowed 18 *L. sakei* pure cultures to be obtained, irrespective of the producer. Of note, *L. sakei* is the key species of lactic acid bacteria in the meat environment (e.g., fermented sausages) [47], hence its presence in the analyzed smoked sausages is not unexpected. However, the studied isolates were obtained from a relatively understudied biological niche, thus representing a potential source of undisclosed biodiversity. *L. sakei* is a psychrotrophic, microaerophilic, facultatively heterofermentative species that is highly adapted to the meat environment through its ability of up-regulating genes encoding for oligopeptide transporters and intracellular peptidases [10]. Furthermore, *L. sakei* has a short lag period and a growth rate that surpasses that of other lactic acid bacteria, thus obtaining a competitive advantage in the meat batter. Additionally, it demonstrated high tolerance to salt, with the ability to thrive in concentrations of up to 6.5 % NaCl [48]. As reported by Wang et al. [49], *L. sakei* can contribute to the loss of nitrite during sausage ripening since, under anaerobic conditions, it possesses the ability to convert nitrite into NO, NO_2_, or N_2_O by nitrite reductase and heme-independent nitrite reductase activity. Moreover, *L. sakei* is able to use ribose in raw meat as carbon source through an ATP-dependent system [50], thus increasing its competitiveness in the food matrix.

Of note, the samples of producer 1 and producer 3 herein studied were subjected to heat-treatment once stuffed into casings, thus potentially reducing the load of pro-technological microorganisms. However, *L. sakei* has already been isolated from cacholeira, a Portuguese blood sausage that is smoked and blanched in hot water for 5 min at about 85 °C [40], thus confirming the negligible effect of a short heat treatment on the viability of this resilient pro-technological species.

In the L. *sakei* studied cultures, no esterase or lipase activity was observed. These results are in accordance with those obtained by Osimani et al. [10] for *L. sakei* isolated from Ciauscolo fermented sausages produced in Italy, and by Ammor et al. [51] for *L. sakei* isolated from traditional dry sausage produced in France. However, esterase in-vitro production has been observed by Amairi et al. [52] in a *L. sakei* strain isolated from a French sausage, suggesting the presence of this enzyme as intracellular protein.

In this study, all the *L. sakei* showed a notable protease activity, irrespective of the producer. In fermented meat products, proteolysis of myofibrillar and sarcoplasmic protein is the result of meat endogenous enzymes and microbial metabolism, with a crucial role of intracellular amino di and tripeptidases of lactobacilli in producing aroma and flavor compounds as low molecular weight peptides and free amino acids [45,53]. The results herein collected were consistent with those obtained by Osimani et al. [10] who detected the activity of leucine and valine arylamidase enzymes in *L. sakei* isolated from Ciauscolo; moreover, a high proteolytic activity was also observed by Li et al. [54] in salted yak meat inoculated with *L. sakei*. Interestingly, Li et al. [54] observed a salt-dependent effect on the proteolytic performance. In more detail, Li et al. [54] reported a reduction of proteolysis resulting from an increase in salt content, hence the high salt tolerance of *L. sakei* confirms its suitability as potential starter or adjunct culture in meat products containing curing salts.

Exopolysaccharides produced by lactic acid bacteria can be classified based on their monosaccharide composition (homologous polysaccharides, containing the same monosaccharide, or heterogeneous polysaccharides, containing different monosaccharides) [55]. Strains of *L. sakei* already showed the capability of producing EPS (e.g., dextran) with potential applications in food processing to improve taste, texture, and stability of food matrices [55].

In the present study, 3 out of the 18 *L. sakei* isolates showed mucoid colonies when grown on MRS agar added with sucrose, thus suggesting EPS production. Interestingly, Hilbig et al. [27] observed positive texture modifications (increase in softness and spreadability) in fat-reduced raw sausages (Teewurst) containing homopolysaccharides produced by *L. sakei*, with no negative alteration of the taste. In this regard, adjunct or starter cultures able to produce EPS should carefully be used based on the desired texture features (e.g., hardness, cohesiveness, springiness, etc.) of the final product.

Interestingly, none of 18 *L. sakei* isolates showed the presence of the *hdcA* gene for histamine production. This result is particularly encouraging since the absence of this gene supports the potential use of the isolates as autochthonous starter or adjunct cultures in processed meat.

Regarding the assays performed to test the *L. sakei* isolates for bacteriocin production, it is likely that none of them harbored the genetic determinants for bacteriocin synthesis, as none of the tested cultures exhibited a bactericidal effect towards *L. innocua*. Consequently, these results will not be discussed further.

Finally, regarding the acidification performance in synthetic growth media, all the isolates allowed a rapid drop of pH (below 4.5) in 24 h to be obtained, irrespective of the addition of curing salts (nitrates or nitrites) and sodium chloride to the MRS broth, thus showing their suitability to be used in meat fermentation processes. In fermented sausages, it is observed that, a rapid acidification of the meat batter is the main requisite to assure the safety of the product. That is, the sooner the meat batter is acidified, the stronger the inhibition of the pathogenic or alterative microflora naturally occurring in the raw meat [56,57].

In this study, one *W. viridiscens* was also obtained. Interestingly, Kameník et al. [14] isolated a *W. viridescens* strain able to survive the heat treatment of hot-smoked dry sausage, thus explaining the presence of this microorganisms in the smoked samples herein analyzed. Interestingly, *Weissella* species isolated from Brazilian artisanal cheese showed proteolytic activity [58], thus confirming the results on protease activity herein observed.

The occurrence of coagulase-negative cocci in the smoked sausages of bovine meat herein studied was also investigated. In all the samples, high counts were observed, irrespective of the producer. As reviewed by Stegmayer et al. [44], coagulase-negative cocci occurring in the meat batter have the ability to influence the hue of the red color, due to the development of nitrosomyoglobin (pink-red) pigment. Of note, nitrate and nitrite salts are employed in the curing of meat products. As reported by dos Santos Cruxen [59], coagulase-negative cocci are able to convert nitrate to nitrite through nitrate reductase activity; moreover, these microorganisms participate in converting nitrite into nitric oxide which promotes the formation of nitrosomyoglobin, thus enhancing the redness of sausages.

In the present study, the counts of coagulase-negative cocci were in accordance with those reported by other studies on fermented or smoked (unfermented) sausages [12,37,60,61]. In addition, the isolation campaign allowed *S. equorum* and *S. casei* pure cultures to be obtained. The species *S. equorum* has already been isolated from French and Italian naturally fermented sausages as well as from salt used in dry-cured ham, as reported by Leroy et al. [62]. This species has also been detected in the staphylococcal ecosystem of kitoza, a traditional Malagasy product manufactured with strips of beef meat [60]. Moreover, biofilms containing *S. equorum* were found on the surfaces of processing equipment for the production of traditional dry fermented sausages [61]. It is known that coagulase-negative cocci with lipase and protease actively contribute in developing the distinctive flavor of fermented meat products; hence, the analysis of lipase and protease activity can be a valuable indicator to screen suitable starter or adjunct cultures [63]. Considering the lipase activity showed by some of the *S. equorum* cultures herein studied, this enzymatic activity has already been observed in *S. equorum* isolates obtained from different high-salt fermented foods [32]. Lipase synthesized by coagulase-negative staphylococci has the ability to break down fatty acids via partial *β*-oxidation, resulting in the generation of free fatty acids that are precursors of aroma volatile compounds as esters, aldehydes, ketones, lactones, and alcohols [64]. As for protease activity, protease-producing strains of *S. equorum*, showing outstanding ability to degrade sarcoplasmic protein, have already been isolated by Ju et al. [65] from low-salt ham. Moreover, a *S. equorum* strain showing the activity of the aminopeptidase leucine arylamidase has been isolated by Li et al. [63] from traditional dry-cured duck. The hydrolytic activity of proteases and peptidases has a pivotal role in the initial breakdown of myofibrillar and sarcoplasmic proteins, furthermore these classes of enzymes are essential for the production of small peptides and amino acids throughout the later stages of ripening [66], thus strongly influencing the final flavor of fermented sausages. Most of the isolates of *S. equorum* herein studied showed nitrate reductase activity. A high nitrate reductase activity has already been reported by Sánchez Mainar & Leroy [67] for *S. equorum* strain used as starter culture in minced meat models. Similarly, Gøtterup et al. [68] observed nitrate reductase activity in *S. equorum* isolated from fermented sausages and used as starter culture in meat model system.

As for *S. casei* (basionym *Staphylococcus succinus* subsp. *casei*), this microbial species has already been detected in salami, and from environmental swabs of meat production plants [69], thus confirming its adaptation to the meat environment. As for enzymatic activities, a few isolates of *S. casei* showed lipase activity. Of note, lipolytic strains of *S. casei* have already been isolated from the traditional fermented food Ngari and from traditional Korean fermented soybean [70]. Moreover, the proteolytic activity of *S. casei* has already been observed in strains isolated from traditional Korean fermented soybean foods [71]. As for nitrate reductase activity, the results herein collected agree with those published by Sánchez Mainar & Leroy [67] who did not observe nitrate reductase for a *S. succinus* strain used as starter culture in minced meat models. A few *S. casei* isolated in the present study showed colonies with mucoid appearance in agar plates containing sucrose. Studies revealed the production of EPS from *S. succinus* isolated from Ngari [72], thus suggesting the ability of the analyzed isolates to produce EPS.

Finally, in the samples herein studied, the absence of pathogenic microorganisms such as *L. monocytogenes*, *Salmonella* spp., *E. coli*, and of hygiene indicators such as Enterobacteriaceae and coagulase-positive staphylococci highlighted the proper application of good manufacturing practices during processing, thus confirming the safety of the product.

## 5 Conclusions

Based on the results, the different production processes applied to the collected samples resulted in differences in the proximate composition as well as in morpho-textural, physicochemical characteristics, and microbial loads. The isolation campaign showed the dominance of the typical key microorganisms occurring in fermented or unfermented sausages (*L. sakei*, *S. equorum*, and *S. casei*), thus suggesting that the heat-treatment applied to samples of producers 1 and 3 was not sufficient in affecting the viability of those resilient bacteria.

The microbial isolates herein studied revealed pro-technological traits, allowing the selection of a few candidates as starter or adjunct cultures for their future use by the meat industry. In more detail, isolates of *L. sakei* S7, S13, and S27 showed a potent in-vitro acidification performance, together with the production of EPS and proteases. Moreover, isolates of *S. equorum* S1 and S2 were characterized for protease and lipase activity, and isolates *S. casei* S21 and S28 showed good lipase activity, protease activity, and the production of EPS. All isolates of *S. equorum* except S2 showed nitrate reductase activity, a pivotal characteristic for the utilization of this species as a starter or adjunct culture.

Further research is needed to test the abovementioned pro-technological features of the isolates in processed meat in-vivo models. Moreover, the application of culture-independent methods, such as next-generation sequencing, could provide deeper insights into the complex microbial communities of artisan smoked bovine sausages.

## Data availability

All data accessed and analyzed in this study are available in the article.

## CRediT authorship contribution statement

**Giorgia Rampanti:** Writing – original draft, Investigation, Formal analysis. **Daniela Nikolovska Nedelkoska:** Writing – review & editing, Investigation. **Tatjana Kalevska:** Investigation, Formal analysis. **Tanja Stojanovska:** Investigation, Formal analysis. **Joanna Harasym:** Writing – original draft, Investigation. **Federica Cardinali:** Investigation, Formal analysis. **Agnieszka Orkusz:** Formal analysis. **Vesna Milanović:** Formal analysis. **Cristiana Garofalo:** Formal analysis. **Alessio Bonifazi:** Investigation, Formal analysis. **Lucia Aquilanti:** Writing – review & editing, Resources. **Andrea Osimani:** Writing – review & editing, Supervision, Resources, Conceptualization.

## Declaration of competing interest

Dear Editor, regarding the manuscript titled “Smoked sausages of bovine meat produced in North Macedonia as a source of pro-technological lactic acid bacteria and coagulase-negative cocci” submitted for publication in Heliyon, the authors declare that they have no known competing financial interests or personal relationships that could have appeared to influence the work reported in this paper.
